# Clinical evaluation of a simple uroflowmeter for categorization of maximum urinary flow rate

**DOI:** 10.4103/0970-1591.32058

**Published:** 2007

**Authors:** Simon Pridgeon, Christopher Harding, Douglas Newton, Robert Pickard

**Affiliations:** * Department of Urology, Freeman Hospital, Newcastle upon Tyne, UK; ^ School of Clinical and Laboratory Sciences, Newcastle University, Newcastle upon Tyne, UK; † School of Education, Durham University, Durham, UK; § School of Surgical and Reproductive Sciences, Newcastle University, Newcastle upon Tyne, UK

**Keywords:** Bladder outlet obstruction, urinary symptoms, uroflowmetry

## Abstract

**Objective:**

To evaluate the accuracy and diagnostic usefulness of a disposable flowmeter consisting of a plastic funnel with a spout divided into three chambers.

**Materials and Methods:**

Men with lower urinary tract symptoms (LUTS) voided sequentially into a standard flowmeter and the funnel device recording maximum flow rate (Q_max_) and voided volume (V_void_). The device was precalibrated such that filling of the bottom, middle and top chambers categorized maximum input flows as <10, 10-15 and > 15 ml s^−1^ respectively. Subjects who agreed to use the funnel device at home obtained readings of flow category and V_void_ twice daily for seven days.

**Results:**

A single office reading in 46 men using the device showed good agreement with standard measurement of Q_max_ for V_void_ > 150 ml (Kappa = 0.68). All 14 men whose void reached the top chamber had standard Q_max_ > 15 ml s^−1^ (PPV = 100%, NPV = 72%) whilst eight of 12 men whose void remained in the bottom chamber had standard Q_max_ < 10 ml s^−1^ (PPV = 70%, NPV = 94%). During multiple home use by 14 men the device showed moderate repeatability (Kappa = 0.58) and correctly categorized Q_max_ in comparison to standard measurement for 12 (87%) men.

**Conclusions:**

This study suggests that the device has sufficient accuracy and reliability for initial flow rate assessment in men with LUTS. The device can provide a single measurement or alternatively multiple home measurements to categorize men with Q_max_ < 15 ml s^−1^.

Measurement of maximum urinary flow rate (Q_max_) is widely used in the assessment of men complaining of lower urinary tract symptoms (LUTS). Although Q_max_ varies with age and voided volume (V_void_), a reduced flow rate can be used clinically to suggest the presence of bladder outlet obstruction (BOO). For clinical categorization cut-off values have been identified whereby men with Q_max_ ≤ 15 ml s^−1^ have an approximate 70% chance of having BOO whilst men with a value > 15 ml s^−1^ have a 65% chance of not having BOO.[1] Standard uroflowmeters differentiate urine weight change to give a continuous plot of flow rate against time which is smoothed by internal electronic filtering to allow precise (± 5%) measurement of Q_max_.[2] Most clinicians use a single office measurement of flow rate as part of their assessment of men with LUTS but this approach may not be ideal because of physiological variation and nonrepresentative V_void_.[3] Approaches to address these deficiencies include obtaining multiple office readings which may improve diagnostic accuracy but is time-consuming and costly[4] or provision of home electronic flowmeters which are also expensive and difficult to maintain.[5] Another possibility is home use of a disposable uroflowmetry device which enables multiple measurements in line with an individual's day-to-day voiding habits and could potentially be used as part of initial assessment of men with LUTS prior to specialist referral.[6] An ideal device would be accurate, simple to use and inexpensive. Prototype devices have been developed but, to our knowledge, are not being routinely used in practice.[6,7] Recently, a simple inexpensive funnel device has been made available which is potentially suitable for repeated measurement of maximum flow rate in the patient's home (Uflow meter™, MDTi Ltd, Wolverhampton, UK). We now describe the results of a clinical study which aimed to determine the accuracy and test-retest reliability of the new device in office and home settings with reference to the current standard of a single Q_max_ office-based reading.

## MATERIALS AND METHODS

### The device

**Figure 1:**
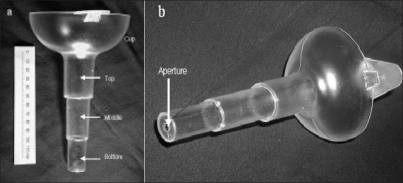
Photographs of the device showing a, the 3-chamber design and b, the 4.6 mm aperture

The flow device consists of a plastic funnel formed from a cup and a spout divided into three chambers with a 4.6 mm diameter aperture placed at the apex [Figure 1]. Fluid poured into the cup will start to fill the funnel as well as flowing out through the aperture. Once inflow (determined by urine flow rate) and outflow (determined by the fixed-diameter aperture and the pressure-head of fluid above it) are equal, a constant maximal fluid level within the funnel will be maintained. With higher filling rates more fluid will be retained in the device and the fluid level will rise in the column provided that the time taken to reach the maximum level (response time) is substantially less than voiding time. Aperture diameter and volume of each chamber are calibrated such that filling of the bottom, middle and top (including cup) chambers corresponds to input flows of < 10, 10-15 and > 15 ml s^−1^ respectively to fit with the current clinical decision-making. The highest chamber reached by urine during the course of a void is recorded by the patient and categorizes their Q_max_ as being within the range for that chamber.

### Patients

Following Institutional and Local Research Ethics Committee approval and with informed consent, we recruited men with LUTS attending for standard office uroflowmetry. Each subject performed two sequential voids in a randomized order either into a standard rotating disc uroflowmeter (Urodyn 1000, Medtronic Ltd., Watford, UK) or into the portable funnel device. The patient held the funnel vertically with the spout above a measuring jug placed on the toilet seat lid to measure flow category and V_void_. For the portable device flow category was derived from the highest chamber reached as observed by the patient and verified by the investigator. The printout from the standard flowmeter was manually read and Q_max_ taken at the highest point of the flow curve discounting spike artefact and with internal filtering set at 10Hz. Participants in the office study who used the portable device successfully and consented to home use were given a device, measuring jug and simple instruction sheet to record flows in a similar manner twice daily for seven days noting Q_max_ category and V_void_ on each occasion.

### Data analysis

In order to assess the level of agreement between continuous data obtained by the standard uroflowmeter against categorical data obtained using the funnel device we assigned the Q_max_ value obtained by standard uroflowmetry to the appropriate category defined on the funnel device (≤10, >10 - ≤15 or >15 ml s^−1^) and then calculated the weighted Kappa statistic (chance corrected correlation coefficient) whereby Kappa > 0.4, > 0.6 and > 0.8 defines moderate, good and excellent agreement respectively. Test-retest reliability of home use of the device was also assessed using the Kappa statistic by comparing all home readings obtained by each individual to the most frequent flow category (mode) documented by that subject. The clinical usefulness of multiple home readings using the funnel device was determined by calculating the sensitivity and specificity of the average home flow against the reference of a single standard office measurement for the categories ≤15 and >15 ml s^−1^. Differences in voided volume were analyzed by paired Student's ‘*t*’ test with significance level set at *P*<0.05.

## RESULTS

### Office observations Subjects

We recruited 46 men with median age 64 (range, 46-82) years, of whom 40 (87%) produced two consecutive flows with voided volume > 150 ml. Most subjects found the funnel device easy to use and read whilst five (11%) had difficulty due to obesity (n=3) or inability to observe the device and void simultaneously (n=2).

### Accuracy

**Figure 2:**
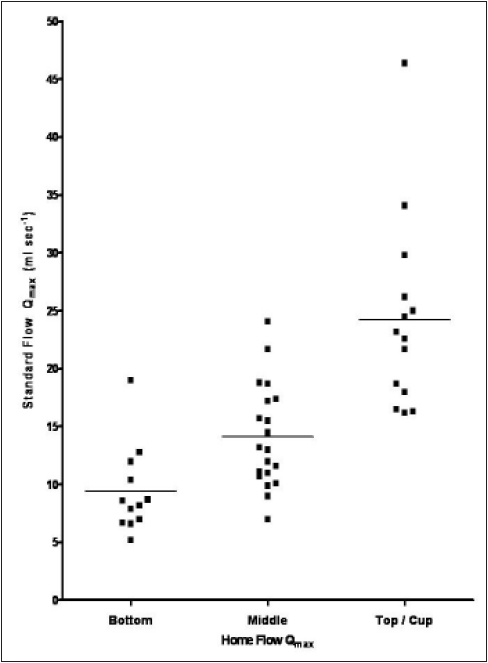
Scatter plot showing the relationship between maximum flow rate (Q_max_) readings obtained in the office using the standard uroflowmetry (vertical axis) and those obtained with the funnel device (horizontal axis). The mean of the single standard office measurements for subjects voiding within each funnel category is indicated by the horizontal bar

Figure 2 compares single measurements of Q_max_ obtained by the standard uroflowmeter and the funnel device. Men whose voids remained within the bottom chamber (< 10 ml s^−1^; n=12) had a mean (SD) Q_max_ with standard uroflowmetry of 9 (4.0) ml s^−1^, whilst voids that reached the middle chamber (10 - 15 ml s^−1^; n=20) or top chamber/cup (>15 ml s^−1^; n=14) had mean (SD) Q_max_ of 14 (4.0) ml s^−1^ and 24 (8.3) ml s^−1^ respectively. All 14 men with office funnel device readings in the top chamber or cup had Q_max_ > 15 ml s^−1^ using standard uroflowmetry (PPV = 100 %) whilst eight of the 10 men with standard Q_max_ < 10 ml s^−1^ were correctly categorized by the device [Table 1]. Overall single office measurement of Q_max_ by the funnel device showed good agreement with standard uroflowmetry (Kappa = 0.61). If data from six men with at least one V_void_ < 150 ml were excluded, the agreement level was improved (Kappa = 0.68). The mean (SD) difference between V_void_ for standard uroflowmeter reading and that for the funnel device reading was −17 (157 ml).

**Table 1:** Accuracy of device in randomized clinic-based comparison with standard uroflowmetry according to Q_max_ thresholds

Funnel category	Standard uroflowmetry Q_max_ (ml s^−1^)			Predictive value (%)	
		
	< 10	10 - 15	> 15	PPV	NPV
Bottom (<10 ml s^−1^)	8	3	1	70	94
Middle (10 - 15 s^−1^)	2	10	8	50	88
Top/cup (>15 ml s^−1^)	0	0	14	100	72

PPV = Positive predictive value; NPV = Negative predictive value

### Home observations Subjects

A total of 14 men with median age 64 (range, 50-81) years used the device at home and all completed the protocol of 14 Q_max_ and V_void_ recordings over seven days. None of these patients reported any difficulty in obtaining recordings using the device at home.

### Accuracy

**Figure 3:**
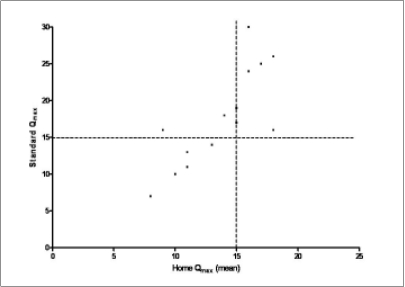
X-Y plot of the averaged notional home Q_max_ (horizontal axis) for 14 men against their single office-based standard reading (vertical axis). The broken lines show a diagnostic cut-off value of 15 ml s-1. Note plots for 12 of 14 (86%) subjects lie in the upper left or lower right quadrants indicating good agreement.

Averaged home readings using the funnel device correctly categorized seven of the nine men with standard Q_max_ > 15 ml s^−1^ (sensitivity = 78%, specificity = 71%) and all five men with a standard Q_max_ measurement ≤15 ml s^−1^ (sensitivity = 100%, specificity = 71%; [Figure 3]). For individual home readings the error in categorization compared to the reference office reading was significantly associated with lower V_void_ [Table 2]. Test-retest reliability using the funnel device was moderate (Kappa = 0.58). The mean (SD) difference between V_void_ for standard test and mean V_void_ for home uroflowmetry within each individual was −54 (67) ml.

**Table 2:** Comparison of the difference in voided volume (V_void_) and the degree of error in estimation of standard single office Q_max_ by home use of the funnel device

	Degree of error of home readings using funnel device compared to single office Q_max_ (number of funnel categories)				

	+2	+1	0	−1	−2
Number of voids	0	3	124	49	20
Mean (SD) difference in V_void_(ml)		−78 (185)	−17 (113)	−68 (139) *P*=0.01	−165 (109) *P*<0.001

Single office flow was assigned to appropriate funnel category and compared to each of the 14 home voids performed by each subject using the funnel device where 0 = same category as office reading, +1 = home reading 1 category higher than office reading, +2 = home reading 2 categories higher than office reading, −1 = home reading 1 category lower than office reading, −2 = home reading 2 categories lower than office reading. The difference in voided volume was calculated as: home Voffice V_void_ - office V_void_

## DISCUSSION

The ageing population and heightened awareness of prostate cancer have increased the number of men with LUTS requesting specialist assessment. This has led many urology practices to set up ‘one stop’ clinics facilitated by asking men to complete symptom questionnaires and frequency-volume charts prior to the office appointment. The addition of ‘home’ uroflow measurement to this preassessment would further streamline the process and help decide management options. The novel device assessed in the present study is potentially suited to this use since it appears to offer acceptable accuracy and reliability with ease of patient use at low cost.

For office use our device showed good agreement with standard measurement, particularly using established diagnostic cut-off values. Most discrepancies occurred due to underestimation by the device of flows in the 15-20 ml s^−1^ range. This may have been partly due to the tendency for lower V_void_ using the device despite randomization or may reflect the known test-retest variation in the standard measurement of Q_max_ (SD = 2 ml s^−1^).[8,9]

The home part of the study was conceived to assess both test-retest reliability of the device and predictive value of multiple recordings compared to a single electronic office measurement. The device showed moderate reliability as indicated by the Kappa statistic which may reflect the known variation of Q_max_ with voided volume and time of void[3] or readings being ‘borderline ‘between two chambers. In common with a previous study using a home-based electronic flowmeter our home device tended to underestimate a standard office measurement of Q_max_.[5] Our data suggest that this may be at least partly related to lower home V_void_ and this again is consistent with previous work.[5,10] Encouragingly, agreement was best for flows ≤ 15 ml s^−1^ where diagnostic decisions will be crucial. If the new device was used at home as a ‘screen’ for onward referral for formal uroflowmetry underestimation of flow rates > 15 ml s^−1^ would be of less concern since this would not reduce test sensitivity for detection of flow ≤ 15 ml s^−1^. Taken together these findings suggest that multiple use of our simple uroflowmetry device does provide a valid and reasonably reliable measure of Q_max_ and that multiple readings using the device could be of potential use in the initial assessment of men with LUTS.

In terms of simplicity the device tested in the present study is intermediate between the very basic ‘Streamtest’ Cup proposed by Currie[7] and the more complicated multi-exit port device of Pel and van Mastrigt.[6] The device showed similar accuracy using different flow thresholds to the ‘Streamtest’ but inferior reliability to the multi-port device which was, however, tested on young noncomplaining volunteers with higher V_void_. We feel that the ability of the funnel device to categorize men into widely used flow rate bands together with its manufacture using a single plastic molding combines low cost [estimated at $8.60 (≤ 5; £ 7.30) per unit] and acceptable accuracy making it suitable for disposable use prior to an office visit for men complaining of LUTS.

The addition of flow data to the widely used frequency volume chart completed prior to the initial office visit would greatly aid diagnosis and treatment planning. In the UK and other countries where all men are initially seen by a family care practitioner it could also be used to help select men for specialist referral. Men with most readings in the upper chamber or cup (notional average flow > 15 ml s^−1^) are unlikely to have BOO and alternative explanations for their symptoms should be sought or else they could be monitored in the community. Those with multiple flows in the middle or lower chambers (≤15 ml s^−1^) could be selected for urology referral and a formal flow assessment if required. Home flow measurements would also be useful for those patients who are unable to provide a representative void during an office visit. We next intend to establish the minimum number of ‘home’ flows required to give the best estimate of standard Q_max_ and then compare patient management with and without clinician use of the data generated.

## CONCLUSION

This simple inexpensive uroflowmetry device allows multiple estimates of Q_max_ to be made in the home setting. The accuracy and reliability of the device appears sufficient to allow categorization around the standard threshold of 15 ml s^−1^ suggesting its usefulness in the preliminary assessment of men with LUTS prior to an office visit.

## Figures and Tables

**Table:** 

1.	Place of publication	:	Mumbai
2.	Periodicity of its publication	:	Quarterly (January, April, July and October)
3.	Printer's Name	:	Medknow Publications Pvt. Ltd.
	Nationality	:	Indian
	Address	:	A-108/109, Kanara Business Center,
			Off Link Rd, Ghatkopar (E),
			Mumbai - 400075, India
			Phone: 91-22-6649 1818/1816,
			Fax: 91-22-6649 1817
4.	Publisher's Name	:	Medknow Publications Pvt. Ltd.
	Nationality	:	Indian
	Address	:	A-108/109, Kanara Business Center,
			Off Link Rd, Ghatkopar (E),
			Mumbai - 400075, India
			Phone: 91-22-6649 1818/1816,
			Fax: 91-22-6649 1817
5.	Editor's Name	:	Nitin S. Kekre
	Nationality	:	Indian
	Address	:	Department of Urology, Christian Medical College,
			Vellore - 632 004, Tamil Nadu, India
			Phone: +91-0416-2282111, Fax: +91- 0416 - 2232035,
			E-mail: editor@indianjurol.com.
6.	Names and addresses of individuals who own the newspaper and partners or shareholders holding more than one per cent of the total capital.	:	Urological Society of India
I, **Nitin S. Kekre**, hereby declare that the particulars given above are true to the best of my knowledge and belief.			
		**Nitin S. Kekre**	
Date: 12^th^ April, 2007		Editor	

## FORM IV

Statement about ownership and other particulars about newspaper (**Indian Journal of Urology**) to be published in the first issue every year after the last day of February
